# Psychosocial Impacts Relating to Dental Injuries in Childhood: The Bigger Picture

**DOI:** 10.3390/dj7010023

**Published:** 2019-03-04

**Authors:** Helen Rodd, Fiona Noble

**Affiliations:** 1 School of Clinical Dentistry, University of Sheffield, Sheffield S10 2TA, UK; h.d.rodd@sheffield.ac.uk; 2 Department of Paediatric Dentistry, Charles Clifford Dental Hospital, Sheffield S10 2SZ, UK

**Keywords:** dental trauma, psychosocial, impacts, children

## Abstract

Traumatic dental injuries (TDI) in childhood are fairly commonplace, with a reported prevalence of up to 30% worldwide. These injuries can have significant impacts on patients, their families and dental professionals; however, this area is currently underrepresented within paediatric oral health research. The psychosocial impacts of traumatic injury are personal to each patient and should be addressed as part of a holistic treatment plan. A review of the current evidence base shows that children who have suffered a traumatic injury to the dentition report worse oral-health-related quality of life. They are also more likely to suffer decreased self-esteem due to their appearance, especially where the injury is not effectively managed. Society (including other children) often judges poorly those with obvious dental disease or anomaly, and with the rising use of social media, these judgements can be made by even greater audiences. There is currently a paucity of qualitative research in this topic to explore the negative psychosocial impacts of dental trauma in greater detail. Although there is growing evidence for the benefit of treatment in improving children’s wellbeing following a TDI, the field of paediatric dental traumatology still has much to learn about young patients’ perspectives, experiences and values.

## 1. Introduction

Sustaining a traumatic dental injury (TDI) in childhood is, unfortunately, a common occurrence, with an estimated 80% of all TDIs occurring before the age of 20 [1]. The reported percentage of children who damage their primary or permanent teeth varies widely depending on the country or population studied, with prevalence figures broadly ranging from 10%–30% [2,3,4]. Clearly, a huge number of children, their families and dental healthcare professionals will experience the impacts of a TDI in one way or another. It is therefore surprising that dental trauma research is underrepresented within paediatric oral health research as a whole [5].

Traditionally, trauma research has adopted a biomedical approach, striving to provide a robust evidence base for clinical management. Patient-reported outcomes and experiences have tended to be overlooked, particularly within clinical trials, and this omission is currently being addressed by the International Association of Dental Trauma [6]. The psychosocial impacts of a TDI are unique to each patient, influencing their treatment preferences, resilience and ultimate recovery. Modern medicine and dentistry now recognise the need to identify and address these psychosocial aspects as part of a more holistic approach to healthcare.

The aim of this position paper is to provide an overview of how TDIs and related treatments affect children themselves, drawing on the contemporary literature and highlighting best practice for child-centred research. A systematic review of the literature is outside the scope of this paper; therefore, the authors recognise that an element of selection bias may have occurred in terms of the references selected. Comments made by children to illustrate points made in this paper are attributed to patients seen in the authors’ unit, during interviews or focus groups in previous research which has received ethical approval and appropriate consent. It is hoped that this insight will be of relevance to clinicians when assessing and managing children with TDIs. Although the focus is on children who sustain injury to their permanent incisors, it should be remembered that trauma to the primary dentition also presents a variety of impacts to younger children and their families, including self-perception, social interaction and functional limitations [7,8,9]. 

## 2. Asking the Right Questions in the Right Way

Two fundamentally different approaches can be adopted for exploring the impacts of TDIs in children, broadly categorised as quantitative or qualitative enquires. Each has its inherent strengths and limitations, but whichever approach is used, it is paramount that young participants are actively engaged and the questions posed to them are relevant and understandable [10]. A systematic review conducted in 2014 suggested that investigators who conduct trauma research may have missed opportunities to involve children in the past [11]. Quantitative research relies on questionnaires or scales, providing numerical data which are conducive to statistical analysis and interpretation but which may fail to uncover novel or more meaningful insights. In contrast, qualitative research seeks to gain a deeper understanding of an individual’s experiences and behaviours using interviews, focus groups or other participatory activities [12].

### 2.1. Findings from Quantitative Research

To date, our knowledge of TDI impacts in children has been largely based on findings from quantitative research. Investigators have used a variety of oral-health-related quality of life (OHRQoL) questionnaires which have been validated for specific age groups and are available in several different languages [9,10,11,12,13]. These instruments essentially seek to measure how much a child’s dental condition (e.g. TDI, caries status, malocclusion, dental anomaly) affects them in their everyday life [13,14]. The most common response format is to ask children to say how frequently (e.g. in the past three months) they have experienced an impact in order to calculate a total OHRQoL score. Questions typically relate to different domains, such as social wellbeing (e.g. school performance, friendships, smiling), oral symptoms (e.g. pain, discomfort), psychological wellbeing (e.g. self-esteem, confidence) and function (e.g. eating, speaking, sports participation).

The first paper to report on TDI-related OHRQoL in children was published in 2002 [15]. This was a school-based, cross-sectional study involving 304 Brazilian children aged 12 to 14 years. Following a clinical exam to record the presence of a TDI, caries or malocclusion, children completed the Oral Impacts on Daily Performance (OIDP) questionnaire. Total OIDP scores were adjusted for caries or a malocclusion as potential confounders. The key finding was that children with an untreated TDI were 20 times more likely to report a negative impact on their daily life compared to their peers. Impacts were recorded in all domains, notably affecting smiling, eating and socialising. Since this landmark study, there have been a number of similar investigations in different populations and countries. A systematic review and meta-analysis published in 2018 identified 26 studies which reported on the OHRQoL of children following a TDI, 16 of which related to injuries in the permanent dentition and involved children aged 8 to 15 years [13]. The studies had been predominantly conducted in Brazil in the period 2011–2015 and involved school-based populations. The key finding was that, despite considerable study heterogeneity, children with a TDI reported significantly worse OHRQoL than their uninjured peers (OR: 1.61; 95% CI 1.16–2.23). Notably, impacts were seen within the social domain.

These cross-sectional studies have provided persuasive evidence that untreated TDIs may have a negative impact on children’s wellbeing as a whole. However, in order to gain greater understanding of what clinical, environmental or personal characteristics may predict OHRQoL within the context of a TDI, more complex study designs and statistical analysis are required. A longitudinal investigation involving 108 children who were treated for a variety of TDIs in a British dental hospital does go some way to meet these criteria [16]. This study collected personal, clinical and psychometric data, including OHRQoL (from the self-report Child Perceptions Questionnaire) at baseline and at six-month follow-up. Structural equation modelling revealed that girls reported poorer OHRQoL than boys, which is in keeping with findings from other studies [17]. Good family support and mixed coping styles appeared to be predictors of better OHRQoL and, interestingly, the severity/complexity of the TDI did not directly predict OHRQoL. Put more simply, children with a minor TDI may actually report worse OHRQoL than a child with a complex TDI. Reassuringly, OHRQoL did improve over a six-month period, although some children continued to report specific functional impacts due to eating difficulties. This was attributed to the fact that many of these young patients were wearing partial dentures following tooth loss or experienced repeated failure of large anterior resin composite restorations. 

### 2.2. Findings from Qualitative Research

The first published narrative to highlight the wider impacts of a traumatic dental injury was actually published over 60 years ago [18]. This was a case report of a 9-year-old boy who attended a British dental hospital having sustained uncomplicated crown fractures of four permanent incisors. As well as detailing the clinical treatment, the authors described the patient’s psychosocial upset from the injury. The boy was an active member of two choirs, but after fracturing his incisors, the choir master noted he ‘lisped’, and the boy lost his place in the choirs. The child’s mother reported that her son had become quiet and moody, experienced disturbed sleep and sibling rivalry. It is remarkable how some of these impacts map so closely with the theoretical model of OHRQoL, proposed many decades later [19,20]. However, since this insightful paper, there has been a paucity of studies that have adopted a qualitative approach to explore the psychosocial aspects of a TDI in more depth. 

## 3. Appearance-Related Concerns

Societal pressures to conform to beauty ‘norms’ are immense for today’s young people. Furthermore, there is a wealth of literature showing that social judgements are commonly made in relation to an individual’s facial or dental appearance [21,22]. These social judgements, negative or positive, may have profound and lifelong consequences in terms of relationship success, career prospects and even judicial outcomes [23]. 

In order to determine whether children make judgements about other children on account of their visible TDIs, investigators have explored attribute ratings ascribed to photographs of child subjects with fractured or discoloured incisors [24,25,26]. These studies have revealed that school children rate photographic subjects with a normal dentition significantly more positively than the same photographic subject with poor incisor aesthetics due to a TDI. The authors concluded that young people appear to make negative social judgements on the basis of visible incisor trauma. Accepting the acknowledged limitations of the experimental design, the clinical relevance is clear: Children should be provided with high-quality and expedient dental treatment for their injuries, which restore aesthetics as well as function. Appearance-related teasing and bullying is an increasing concern, undoubtedly fuelled by the ubiquitous use of social media by young people [27]. Children with a compromised dental appearance as a result of a TDI may worry about receiving unkind comments.

‘I was nervous and worried that my teeth would look a mess and people would laugh or stare at me.’

*(Sophie, 14-year-old girl)*

Furthermore, anxieties can be heightened at important life events, such as moving to a different school [28]. Clinicians should therefore be empathetic and alert to each child’s social circumstances and endeavour to provide timely treatment to restore incisor appearance and function.

## 4. Other Psychological Impacts

One aspect of dental trauma which may not have received sufficient consideration is the nature or circumstances of the ‘accident’ itself and the potential for children to subsequently experience mental health problems. On looking at the wider trauma literature, there are some interesting data on the short- and long-term psychological impacts of sustaining a physical injury, irrespective of what it is. A UK study of around 100 children attending an emergency department following a road traffic accident explored anxiety, depression, post-traumatic stress disorder (PTSD) and coping styles at six weeks and eight months after the physical injury [29]. The key finding was that almost half of the children showed evidence of psychological distress six weeks after the accident, and after eight months, around one in six continued to suffer from PTSD. Interestingly, there was no correlation between the severity of the physical injury itself and the degree of subsequent emotional distress. Paediatric emergency teams are now calling for better psychological assessment of children so that support can be put in place to emotionally support those at risk of PTSD [30]. It is clearly important that dental health professionals are also aware of the potential for some children with a TDI to experience some degree of psychological distress from the accident itself, be it a road traffic accident, sporting event or assault. Mental health sequelae may manifest at the child’s subsequent dental visits as anxiety or treatment avoidance, and this should be explored further in future research.

It should also be recognised that children not only have to deal with the accident from their perspective but are also exposed to the distress and other emotions of their friends or parents. A 9-year-old boy describes the aftermath of his bicycle injury as follows:

‘I just saw blood everywhere, I couldn’t think what I had done. Most of my mates were crying… they were all crowded around me. It was horrible.’

The boy’s mother continues to remember the accident:

‘His arm was shaped like a banana but once I realised he wasn’t going to die and could walk, I noticed he has made a real mess of his face, It was all bleeding, they didn’t know where the blood was coming from……..It was horrific, I can’t block it out, I will never forget it.’

A quote from another mother of a 9-year-old boy who sustained a TDI also illustrates the distress children may feel because their parents are upset:

‘And Sam said “is my dad really upset because I’ve lost my teeth?” We said we’d rather he broke his arm, at the end of the day that can be fixed.’

Although the focus of this present paper is the child and not the parent, it should be noted that a number of previous studies have explored the wider impacts experienced by the family following their child’s injury. It is clear that there are high levels of guilt and worry (about the long term success and cost of related treatment) that can impact upon the family unit as well as children themselves [31,32,33]. Parents may make comments or display behaviours which are not helpful for a child’s emotional recovery from a dental injury. Indeed, previous research has shown that good family support is an important predictor of OHRQoL outcomes in children following a TDI [16].

## 5. The Effect of Trauma-Related Treatment

The final aspect to consider is the impact on children from TDI-related treatment. The high demands placed on young patients and their family unit from prolonged courses of treatment for complex TDIs have been reported by a couple of studies, involving relatively small numbers of children, in the United Kingdom, Canada and North America [31,32,33,34]. In terms of treatment burden, children frequently have to miss school or other social and sporting activities in order to travel long distances to specialist paediatric dentistry services.

‘I got behind with my school work as I had to go to the dental hospital frequently. I know it’s worth it though.’

*(Sophie, 14-year-old girl)*

Investigators at Torronto Children’s Hospital, Canada, conducted a fascinating study to explore child and family impacts following an avulsion injury [31]. In the first year following the avulsion and tooth replantation, children attended an average of nine visits, including at least one emergency unplanned visit, received over seven hours of dental treatment and missed an estimated two weeks of schooling. Ultimately, 18% of replanted teeth were extracted in that first year. Around one-third of children stated that, in retrospect, they would have preferred not to have had the tooth replanted. The authors surmised that children should be more actively involved in decision-making when replanting teeth of very poor prognosis. A conference paper published in 2009 appraised the supporting literature and highlighted a number of treatment-related impacts experienced by children and their families, including dental anxiety, dental avoidance, financial burden and dissatisfaction with care [34]. 

Nonetheless, there is evidence that children who receive treatment for a TDI report significantly improved OHRQol in the short term, although they may still report some social and functional impacts [16,35,36]. The previously mentioned case study of the 9-year-old boy described the positive psychosocial outcomes following restoration of his four fractured incisors [18]:

‘After treatment his whole outlook became lighter and he began to assume the confidence previously lacking. His appetite improved there was a gain in weight and he now slept well. His position in the family was re-established and shortly afterwards he was reinstated as the choir soloist and he has been successful in his scholarship to a public school.’

Clearly, this is just one child’s experience, and there remains a need for high-quality research to better inform service providers and commissioners of the psychosocial impact of dental interventions for children with a TDI. Indeed, there is considerable scope to include population-based assessments of psychosocial impacts in larger-scale child epidemiological surveys. Furthermore, enquiries need to consider the longer-term implications throughout adolescence and early adulthood, when aesthetics may be further compromised and poor prognosis teeth may be ultimately lost [37]. 

## 6. Summary

In summary, it is hoped that this overview has provided some insight into the wide-ranging impacts that TDIs may have on children. Although there is growing evidence for the benefit of treatment in improving children’s wellbeing following a TDI, the field of paediatric dental traumatology still has much to learn. Priority should be placed on developing appropriate patient-reported outcome measures to allow a more meaningful evaluation of the care we provide. Further qualitative research is also required, particularly with adolescents, to inform clinicians about young patients’ perspectives, experiences and values and how these may change over the course of treatment and indeed the life course. 
